# Can we extend the prolonged effects of a 180-s stretching exercise by applying an additional 15-s stretching bout?

**DOI:** 10.3389/fspor.2024.1473746

**Published:** 2024-11-07

**Authors:** Masatoshi Nakamura, Kazuki Kasahara, Yuta Murakami, Kosuke Takeuchi, Ewan Thomas, Antonino Scardina, Andreas Konrad

**Affiliations:** ^1^ Faculty of Rehabilitation Sciences, Nishi Kyushu University, Kanzaki, Japan; ^2^ Institute for Human Movement and Medical Sciences, Niigata University of Health and Welfare, Niigata, Japan; ^3^ Department of Physical Therapy, Kobe International University, Kobe-shi, Japan; ^4^ Sport and Exercise Sciences Research Unit, Department of Psychology, Educational Science and Human Movement, University of Palermo, Palermo, Italy; ^5^ Institute of Human Movement Science, Sport and Health, University of Graz, Graz, Austria

**Keywords:** range of motion, tissue hardness, pain pressure threshold, static stretching, knee extensors

## Abstract

**Introducation:**

This study aimed to clarify whether or not the prolonged effect of a 180-s static stretching (SS) exercise could be maintained for a longer period by performing an additional short-term (15-s) SS exercise following a 15-min rest.

**Methods:**

The participants were 15 healthy untrained adult males in three conditions: (1) SS condition (180-s SS exercise only); (2) SS + 15 s condition (180-s SS and 15-s SS exercise during the rest period); and (3) noSS + 15-s condition (15-s SS exercise only). The knee flexion range of motion (ROM), pain pressure threshold (PPT), and tissue hardness were measured before (PRE), immediately after (POST), and 15 min and 30 min after the SS exercise.

**Results and discussion:**

Significant interaction effects were observed in all variables. *Post hoc* tests showed that knee flexion ROM showed an immediate significant change (*P* < 0.05) and also at 15 min and 30 min after the SS exercise in the SS + 15 s condition. Tissue hardness showed an immediate significant decrease (*P* < 0.05) and also at 15 min, but not at 30 min after the SS exercise in the SS + 15 s condition. The additional short-term (15 s) SS exercise in the rest period may have a potential long-lasting effect on ROM increase and tissue hardness decrease.

## Introduction

Static stretching (SS) exercise is performed as part of a warm-up routine to prevent sports injuries. In particular, chronic SS intervention can effectively prevent muscle strain (1, 2). A single bout of SS exercise can increase the range of motion (ROM) (3–5) and decrease passive stiffness of the muscle-tendon unit or muscle (6–9).

In addition to acute effects, sustained effects (i.e., prolonged/time-course effects) are also important in sports settings. Previous studies have reported that ankle dorsiflexion (DF) ROM increased immediately after all stretching durations but returned to baseline after 10 min (10). In addition, regarding the change in passive stiffness of the ankle plantar flexors (11), passive stiffness decreased following a 2-min SS exercise but returned to baseline value after 10 min. In the 4- and 8-min SS exercise durations, the change in passive stiffness returned to baseline value after 20 min. Otherwise, Sato et al. (12) found no significant change in the shear elastic modulus of the medial gastrocnemius muscle after 20 s of SS exercise, but DF ROM increased up to 10 min after. In addition, Mizuno et al. (13) conducted a detailed study of the prolonged effects of a 5-min SS exercise on ankle plantar flexors. Interestingly, they reported that the increase in DF ROM lasted for up to 30 min, while the decrease in muscle stiffness returned to baseline within 15 min. In addition, previous studies by Konrad et al. (14) and Konrad and Tilp (15, 16) investigated the acute and prolonged effects of a single bout of 1-, 3-, and 5-min SS exercise on ankle plantar flexors and showed that the decrease in passive stiffness returned to baseline value faster than the increase in DF ROM. These results suggest that the duration of these changes in ROM and passive stiffness is short, and they can return to baseline values in a short time. Also, previous studies suggested that increasing ROM after SS exercise could contribute to a change in pain sensation, so-called stretch tolerance (17–19). Thus, the change in stretch tolerance may be related to the gap in the time course of changes in ROM and passive stiffness.

As described above, the prolonged effect of SS exercise may not last longer than 30 min, which is not sufficient to apply to actual sports situations. In addition, these previous studies were conducted at rest after SS exercise, which is different from a sports setting. In practice, short SS exercise could be performed during sports activities or after longer-duration SS exercise. However, as mentioned above, previous studies investigating the prolonged effects of a single bout of SS exercise have only performed a rest duration without, e.g., applying further stretches to prolong the effects to a longer duration. Therefore, there is a need to investigate whether an additional short-term SS exercise can prolong the effects of SS exercise for a longer period of time, as could be achieved in a sporting environment. This study aimed to clarify whether or not the prolonged effect of 180-s SS could be maintained for a longer period by performing an additional short-term (15 s) SS exercise following a 15-min rest.

## Methods

### Experimental design

**Figure 1:**
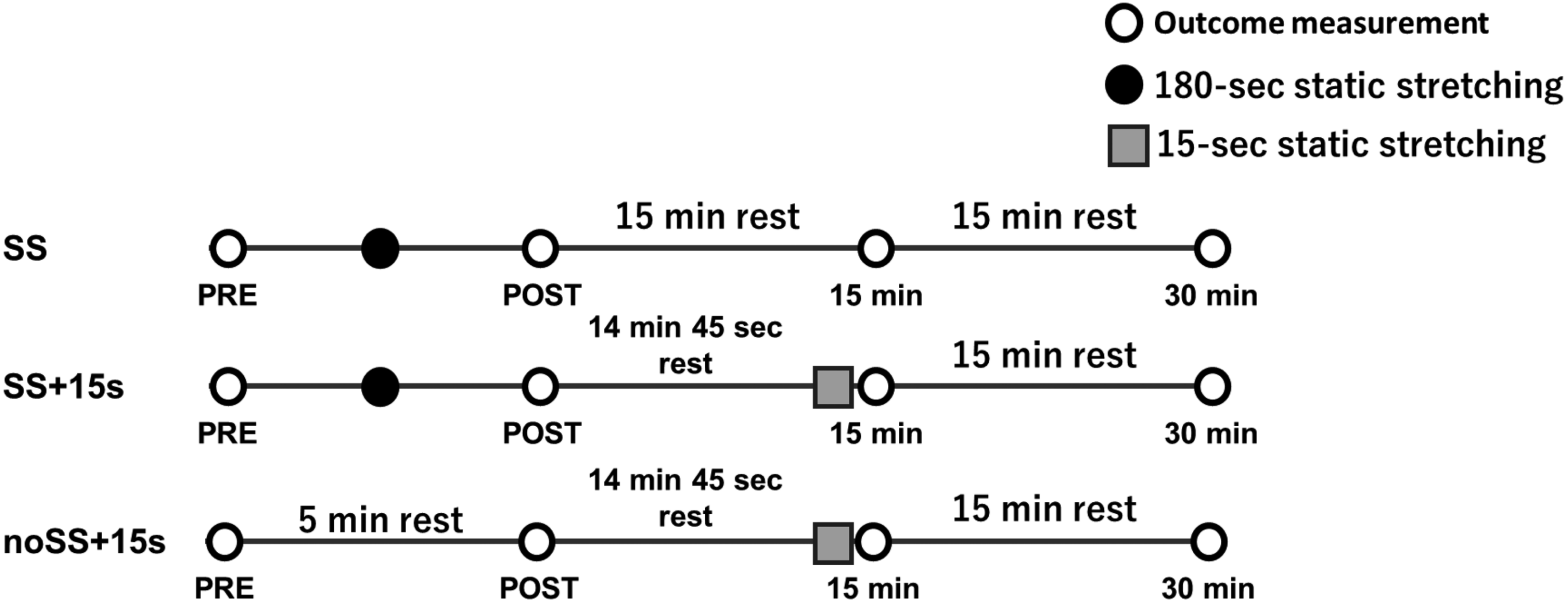
Experimental set-up. All outcome measurements were performed before (PRE), immediately after (POST), and 15 min and 30 min after 60 s × 3 repetitions (180-s) of static stretching (SS) of the knee extensors in SS and SS + 15 s conditions. In the noSS + 15 s condition, participants were measured before (PRE) and after (POST) 300 s of rest in a sitting position. In the SS + 15 s and noSS + 15 s conditions, the 15-s SS was performed just before the 15-min measurement. In all conditions, subjects spent the rest period in a sitting position.

The study was conducted as a randomized repeated-measures controlled experiment. Participants were instructed to visit the laboratory three times, with an interval of at least ≥48 h. Participants were exposed to three conditions: SS, SS + 15 s, or noSS + 15 s (Figure 1). Outcomes were measured in each condition before (PRE), immediately after (POST), and at 15 and 30 min after the SS exercise. Knee flexion ROM, pain pressure threshold (PPT), and tissue hardness were measured in the knee extensors on the dominant side (20). In the SS and SS + 15 s conditions, participants performed three sets of SS for 60 s each on the knee extensors in the dominant leg. The rest between sets was 30 s. Also, SS was conducted 60 s after PRE measurement. In the noSS + 15 s condition, participants performed 300 s of rest to match the time of the SS and SS + 15 s conditions. In addition, in the SS + 15 s and noSS + 15 s conditions, the 15-s SS exercise was performed immediately before the 15-min measurement, in order to investigate the effect of an additional SS exercise.

### Participants

Fifteen healthy, recreationally active males participanted in this study [mean ± standard deviation (SD): age, 22.4 ± 1.1 years; height, 171.3 ± 5.0 cm; weight, 63.0 ± 6.3 kg]. Participants with a history of neuromuscular disease or musculoskeletal injury involving the lower extremities were excluded. The required sample size for a repeated-measures two-way analysis of variance (ANOVA) [effect size = 0.25 (medium, based on the interaction effects for 2-way ANOVA), *α* error = 0.05, and power = 0.80] based on our previous study's effect size about change in ROM immediately after SS exercise (12) using G* power 3.1 software (Heinrich Heine University, Dusseldorf, Germany) was determined to be　more than 10 participants.

 Participants were thoroughly informed about the procedures and objectives of the study, after which they provided written informed consent. The study adhered to the requirements of the Declaration of Helsinki and was approved by the Ethics Committee of Niigata University of Health and Welfare (Procedure # 18561).

### Outcome assessment

#### Knee flexion ROM

Participants were positioned in a side-lying position on a massage bed, with both the hips and the knee of the non-dominant leg flexed at 90° to stabilize the pelvis and prevent any pelvic movement (20, 21). The certified physical therapist (i.e., the primary investigator) guided the dominant leg into full knee flexion while maintaining the hip joint in a neutral position. The knee flexion ROM was operationally　defined as the maximum knee flexion angle just before the onset of discomfort or pain repaorted by participants. Measurements of knee flexion ROM were obtained using a goniometer (MMI universal goniometer Todai 300 mm; Muranaka Medical Instruments, Co., Ltd., Osaka, Japan). We measured the knee flexion ROM three times in each measurement period, and the average value at each measurement period was calculated for further analysis. In addition, none of the participants in this study had their heels touching their buttocks when measuring knee flexion ROM.

#### Pain pressure threshold (PPT)

PPT measurements were carried out with paticipants in a supine position using an algometer [NUTONE TAM-22(BT10); TRY-ALL, Chiba, Japan]. The measurement site was determined at the midpoint between the anterior superior iliac spine and superior border of the patella on the dominant side, targeting the rectus femoris muscle. Gradual pressure was applied through the algometer's metal rod to compress the soft tissue at the designated site. The participant was instructed to press a trigger as soon as they sensed pain, differentiating it from just pressure. The recorded value at this threshold (measured in kilograms per square centimeter) represented the PPT. For each condition, PPT was assessed three times at each measurement period, and the mean of these values was used for further analysis.

#### Tissue hardness

Tissue hardness was assessed using a portable tissue hardness meter (NEUTONE TDM-N1; TRY-ALL Corp., Chiba, Japan), with the participant maintaining the same supine position as for the PPT measurements. This device measured the pushing force until a 14.71 N (1.5 kgf) pressure was reached (22). The participant was instructed to remain relaxed throughout the measurement We measured tissue hardness three times at each measurement period, and the mean value at each measurement period was used for the further analysis.

#### Static stretching (Ss)

SS exercise was performed in a similar manner to the knee flexion ROM assessment). The knee joint was passively flexed to the maximum knee flexion ROM, with participants positioned in a side-lying position. The primary investigator performed three 60-s bouts of SS with a 30-s rest interval between bouts. In the SS + 15 s and noSS + 15 s conditions, a 15-s SS exercise was performed in a similar manner, just before the 15-min assessment. Participant was instructed to be relaxed and keep their torso upright during stretching.

#### Statistical analysis

All statistical analyses were performed using SPSS (version 29.0, SPSS Japan Inc., Tokyo, Japan). To ensure consistency of PRE (baseline) values, a repeated one-way ANOVA was conducted across all conditions. Test-retest reliability for each measure was assessed by calculating the intraclass correlation coefficients (ICCs) from the PRE values.For all the variables, a two-way repeated-measures ANOVA using two factors (test time [PRE vs. POST vs. 15 min vs. 30 min] and conditions [SS vs. SS + 15 s vs. noSS + 15 s]) was used to analyze both interaction and main effects. Where applicable, *post hoc* analyses were conducted using multiple comparison tests with Bonferroni correction to evaluate the differences between PRE and POST and 15 and 30 min. Effect size (ES) were calculated as the difference in the mean value divided by the pooled SD between PRE and POST and 15 and 30 min in each condition. An ES of 0.00–0.19 was considered as trivial, 0.20–0.49 as small, 0.50–0.79 as moderate, and ≥0.80 as large (23). A significance level of 5% was setted, and all the results are shown as mean ± SD.

## Results

Table 1 lists the changes in knee flexion ROM, PPT, and tissue hardness in all conditions. There were no significant differences in any of the PRE variables between the three conditions. The ICC values for knee flexion ROM, PPT, and tissue hardness were 0.98, 0.77, and 0.910, respectively. Significant (*P* < 0.01) interaction effects in knee flexion ROM (*F* = 23.6, *η*_p_^2^ = 0.628), PPT (*F* = 3.68, *η*_p_^2^ = 0.208), and tissue hardness (*F* = 8.23, *η*_p_^2^ = 0.37) were revealed. The *post hoc* test results showed that knee flexion ROM increased significantly (*P* < 0.05) immediately after and at 15 min and 30 min after the SS exercise in the SS and SS + 15 s conditions. In addition, in the noSS + 15 s condition, the 15-min value was significantly (*P* < 0.01) higher than the PRE value (*d* = 0.22).

**Table 1:** Time-course changes (mean ± SD) in knee flexion range of motion (ROM), pain pressure threshold (PPT), and tissue hardness of the knee extensor before (PRE), immediately after, and 15 min and 30 min after the static stretching (SS) exercise.

	Condition	PRE	POST	15 min	30 min	Interaction effect
Knee flexion ROM (°)	SS	133.0 ± 4.4	136.2 ± 4.5*	134.5 ± 4.7*	133.6 ± 4.5*	*P* < 0.001
		*d*=	0.71	0.33	0.13	
	SS + 15 s	132.9 ± 4.3	136.7 ± 3.9*	135.6 ± 4.0*	134.1 ± 4.1*	*F* = 23.6
		*d*=	0.92	0.65	0.27	
	noSS + 15 s	133.3 ± 4.2	133.3 ± 4.4	134.3 ± 4.5*	133.4 ± 4.5	*η_p_*^2^ = 0.628
		*d*=	0.00	0.22	0.01	
PPT (N)	SS	4.1 ± 0.9	4.6 ± 1.1	4.3 ± 1.1	4.2 ± 1.1	*P* < 0.001
		*d*=	0.41	0.16	0.12	
	SS + 15 s	3.9 ± 1.0	4.6 ± 1.3*	4.2 ± 1.0*	4.1 ± 1.2	*F* = 3.68
		*d*=	0.57	0.24	0.22	
	noSS + 15 s	3.8 ± 0.9	3.8 ± 0.8	4.0 ± 0.9	4.1 ± 0.9	*η_p_*^2^ = 0.208
		*d*=	0.05	0.27	0.32	
Tissue hardness (a.u.)	SS	15.5 ± 2.2	13.5 ± 2.3*	14.0 ± 2.4*	14.9 ± 2.6	*P* < 0.001
		*d*=	−0.87	−0.64	−0.24	
	SS + 15 s	15.7 ± 2.8	13.5 ± 2.6*	13.9 ± 2.6*	14.9 ± 2.5*	*F* = 8.23
		*d*=	−0.77	−0.69	−0.31	
	noSS + 15 s	15.6 ± 3.0	15.2 ± 2.8	14.4 ± 3.2*	14.9 ± 2.9	*η_p_*^2^ = 0.37
		*d*=	−0.13	−0.40	−0.26	

The repeated-measures two-way analysis of variance results [interaction effect; *F*-value and partial *η*^2^ (*η_p_*^2^)] are shown in the right column. The Cohen's d effect size (*d*) is also provided from the PRE values for all conditions.

* Significant (*P* < 0.05) difference from the PRE value.

PPT showed a significant increase (*P* < 0.05) immediately after and 15 min after, but not 30 min after the SS exercise in the SS + 15 s condition. However, there were no significant changes in both the SS and noSS + 15 s conditions. Tissue hardness value in the SS condition decreased significantly (*P* < 0.05) immediately after and 15 min after, but not 30 min after the SS exercise (*P* = 0.062, *d* = −0.24). In the SS + 15 s condition, tissue hardness values decreased significantly (*P* < 0.05) immediately after and 15 min and 30 min after the after SS exercise. In the noSS + 15 s condition, the 15-min value was significantly lower (*P* < 0.01) than the PRE value (*d* = −0.40).

## Discussion

In this study, we investigated if the effect of an additional 15-s SS exercise could extend the duration of the effect of a 180-s SS exercise. The 180-s SS exercise increased knee flexion ROM, and the effect was prolonged up to 30 min later. On the other hand, the additional 15-s SS exercise during a rest period increased PPT and decreased tissue hardness for longer. To the best of our knowledge, this is the first study to investigate the effect of an additional short SS exercise on the prolonged effect of SS exercise.

As shown in Table 1, knee flexion ROM increased significantly, and the effect was prolonged up to 30 min. This result expanded on our previous study investigating the time-course change in knee flexion ROM after 180-s SS exercise (39), and showed that knee flexion ROM increased up to 30 min after the 180-s SS exercise. Interestingly, at 15 min after the SS exercise, the ES was small (*d* = 0.33) in the SS condition, whereas it was moderate (*d* = 0.65) in the SS + 15 s condition. The 15-s SS exercise also significantly increased knee flexion ROM in the noSS + 15 s condition (ES = small, *d* = 0.22). Previous studies investigating the acute effect of short-duration SS, such as 15–20-s SS exercise, could increase ROM (12, 24, 25), and the results of this study supported these previous studies. These results indicate that an additional 15-s SS exercises during the rest period increases the effect on knee flexion ROM. In addition, although not significant, the SS + 15 s condition (*d* = 0.27) increased by a little larger magnitude of change than the SS condition (*d* = 0.13), even after 30 min.

Similar to the changes in knee flexion ROM, our results also showed that PPT and tissue hardness changes were significantly altered in the SS + 15 s condition, although the time-course changes of these variables differed. For tissue hardness, although the mechanism of change in tissue hardness after SS exercise is unknown in this study, the shear modulus (the index of muscle stiffness) of rectus femoris has been reported to decrease after a 180-s SS exercise (26). Tissue hardness can reflect changes in subcutaneous tissues, aponeurosis, and muscles and is not the same as muscle stiffness. However, the decrease in tissue hardness could reflect the change in muscle stiffness. In the present study, we extended this finding. Furthermore, SS exercise may decrease tissue hardness through thixotropic changes and increase tissue perfusion (27). We found that a 180-s SS exercise continued to decrease tissue hardness up to 15 min after the SS exercise, and an additional 15-s SS exercise continued to decrease tissue hardness up to 30 min after the SS exercise in the SS condition. However, it was also clear that a short SS duration, such as an additional 15-s SS, would not significantly decrease tissue hardness. It will be necessary to investigate the effects of adding longer SS durations in the future study.

In terms of PPT change, the additional 15-s SS exercise led to a continued increase PPT. Although the detailed mechanism underlying the significant increase in PPT remains unclear in this study, it is plausible that alterations in pain perception resulting from the SS exercise contributed to the observed effect. Behm and colleagues (28, 29) have suggested that hypoalgesic responses could be explained by the gate control theory (30) and the diffuse noxious inhibitory control mechanism (31), both of which are theories of pain inhibition. In line with these hypotheses, the additional 15-s SS exercise appears to have continued a sustained increase in PPT, which persisted for up to 15 min after the exercise.

It has also been reported that the increase in ROM with SS exercise is associated with changes in the passive mechanical property (i.e., muscle stiffness) and stretch tolerance (13, 17, 32). Although the mechanism of the increase in knee flexion ROM seen in this study is unclear, given the changes in tissue hardness and PPT, it is likely that changes in both are associated with an increase in knee flexion ROM.

Previous studies have pointed out that poor ROM (33, 34) and increased muscle stiffness (35, 36) are risk factors for sports injuries. In this study, we examined the effect of an additional short-term SS exercise in addition to a 180-s SS exercise. The results showed that the short-term SS exercise (15-s SS exercise) increased knee flexion ROM and decreased tissue hardness, and that the effect could be prolonged for a longer period (at least up to 30 min). This information is expected to be important for athletes and coaches to increase and sustain the benefits of SS exercise obtained during warm-up.

There were some limitations in this study. The first limitation is the target participants. The study examined the effects on healthy male university students, so it is unclear whether similar results could be obtained for athletes. The second is the duration of the SS exercise, because SS exercise has been suggested to have a dose-response relationship (37). Our results showed that we did not conduct the statistical comparisons in the dependent variables between the conditions, and that there appears to be no difference in the dependent variables at 30 min between the conditions. In addition, shorter stretching durations are often employed in sports settings (12, 38). Therefore, it will be necessary to investigate the effects of an additional stretching exercise after longer and/or shorter stretching durations.

## Conclusion

In conclusion, our results revealed that the 180-s SS exercise increased knee flexion ROM and that the effect of this exercise lasted for 30 min, regardless of whether or not short-term (15-s) SS exercise was added. Interestingly, an additional 15-s SS exercise in the rest period may have a potential long-lasting effect on PPT increase and tissue hardness decrease. These results will provide useful information for athletes and coaches to reconsider warm-up routines.

## Data Availability

The raw data supporting the conclusions of this article will be made available by the authors, without undue reservation.
